# Family incidence of endometriosis in first-, second-, and third-degree relatives: case-control study

**DOI:** 10.1186/1477-7827-8-85

**Published:** 2010-07-11

**Authors:** Kazem Nouri, Johannes Ott, Birgitt Krupitz, Johannes C Huber, Rene Wenzl

**Affiliations:** 1 Department of Gynecologic Endocrinology and Reproductive Medicine, Medical University of Vienna, Vienna, Austria; 2 LKH Gmunden, Upper Austria, Austria; 3 Department of Gynecology and Gynecologic Oncology, Medical University of Vienna, Vienna, Austria

## Abstract

**Background:**

Initial publications examining the hereditary aspects of endometriosis appeared in the early seventies and demonstrated an up to seven-fold risk for endometriosis in first-degree relatives of endometriosis patients. The aim was to evaluate the influence of hereditary aspects on the endometriosis risk in our patient collective.

**Methods:**

In a retrospective cohort study we evaluated the incidence of endometriosis among first-, second-, and third-degree relatives of endometriosis patients and compare it with its incidence among first-, second-, and third-degree relatives of patients without endometriosis.

**Result(s):**

Eighty patients in whom endometriosis had been confirmed laparoscopically and histologically by biopsy and 60 patients in whom no endometriosis had been found during laparoscopy were given a questionnaire about the presence of symptoms associated with endometriosis and its family incidence. Patients of both the endometriosis and the control group were 37.7 ± 6.2 and 45.9 ± 12.0 years of age at the time of the interview, respectively (p < 0.05). Information about the presence of endometriosis was more readily available for relatives of those in the endometriosis group than for those in the control group (325/749 [43.4%] vs. 239/425 [56.2%], p < 0.05). In 5/136 (3.7%) and 8/134 (6.0%) first-degree relatives of endometriosis patients and the control group, respectively, information about the presence of endometriosis was not available (p = 0.554). Endometriosis was found in 8/136 (5.9%) first-degree relatives of patients and in 4/134 (3.0%) first-degree relatives of controls in the real-case analysis (p = 0.248). When comparing endometriosis characteristics between endometriosis patients with and without a history of familial endometriosis, no significant differences were found.

**Conclusion(s):**

There is a trend toward an increased familial incidence of endometriosis. In contrast to the literature, we found a less dramatic increase in familial risk for the development of endometriosis.

## Background

Endometriosis is one of the most common gynecological diseases, and occurs in 2-10% of women of reproductive age [1]. Symptoms include severe pelvic pain, dysmenorrhoea, bladder and bowel discomfort, and infertility. To date, very little is known about its etiology and pathogenesis. Because a number of studies have demonstrated an increased risk for developing the disease in mothers and/or sisters of patients, endometriosis likely has a genetic basis [2-6].

Initial publications examining the hereditary aspects of endometriosis appeared in the early seventies [3]. In 1981, Simpson et al. were able to demonstrate that the risk for endometriosis in first-degree relatives of endometriosis patients is seven-fold compared to the normal population [4]. Five years later, only a four-fold increased risk for developing the disease was estimated for mothers and sisters of an endometriosis patient. [5]. A study conducted in twins demonstrated that the incidence of endometriosis in monozygotic twins was twice that in dizygotic twins [6]. In addition, it has been shown that the severity of endometriosis is higher among patients with a positive family history [7]. Accordingly, one could conclude that if a woman has endometriosis, the risk that her first-degree relatives will also have endometriosis could be anywhere from 4-7 times higher than that of the general population [4,5].

For practical reasons, only a few studies have dealt with the endometriosis incidence in second- and third-degree relatives of endometriosis patients [8]. The purpose of this study was to evaluate the risk of familial endometriosis among first-, second-, and third-degree relatives of women with and without laparoscopically diagnosed endometriosis.

## Methods

The *primary objective *was to evaluate genetic predisposition for endometriosis in a group of patients in whom endometriosis had been confirmed by laparoscopy and biopsy (the study group), and to compare these results to patients in whom endometriosis had been ruled out by laparoscopy (the control group). All procedures were carried out according to the "Good Scientific Practice" guidelines set forth by the Medical University Vienna based on the Helsinky declaration. Informed consent was obtained from all participants.

### Patient collective

In a retrospective control-group study, all infertile women who underwent gynecologic laparoscopy at the Department of Obstetrics and Gynecology of the Medical University of Vienna, Vienna, Austria, between January 2003 and December 2004 were included. Of a total of 318 patients, 178 patients (56.0%) had to be excluded for the following reasons: 163 patients were not willing to participate in the study, or their contact addresses and phone numbers were missing. In 15 patients, the endometriosis was diagnosed only by laparoscopy without biopsy and histological confirmation, and thus, these patients could not be reliably assigned to one of the two groups.

The remaining 140 patients (44.0%) were assigned to one of the two study groups: patients in whom endometriosis had been confirmed laparoscopically and histologically by biopsy (n = 80, endometriosis group); and patients in whom no endometriosis had been found during laparoscopy (n = 60, control group). Patients in the control group had been operated for various indications that are listed in Table 1.

**Table 1:** Indications for gynecologic laparoscopy in the control group (n = 60)

uterine leiomyoma	25 (41.7%)
ovarian cysts or polycystic ovarian syndrome	24 (40.0%)
hydrosalpinx	8 (13.3%)
uterine malformations	2 (3.3%)
ovarian mass	1 (1.7%)

### Study design

#### *Clinical endpoints and parameters*

In the course of a retrospective chart review, we focused on the presence and severity of symptoms associated with endometriosis, including dyspareunia, chronic pelvic pain, infertility, dysmenorrhea and pain at defecation. Endometriosis was classified according to the revised severity score of the American Fertility Society (rAFS) classification.

Patients were contacted by telephone and, using the self-developed questionnaire, asked about gynecologic diseases in all first-degree relatives (i.e., mother, sisters, and daughters), as well as in all second- and third-degree relatives (aunts, cousins). Information about daughters was included as of their 17^th ^year of age onward since endometriosis is known to be hormonally influenced. The maternal and the paternal lines of relatives were evaluated separately. Information about grandmothers was excluded from the study, since reliable information about their medical history could only be provided by a minority of patients. We focused on the following points when requesting relatives' medical history: gynaecologic operations and possible detection of endometriosis; and symptoms such as chronic pelvic pain, infertility, and dysmenorrhea. If the interviewees were not aware of this information, the answer on the questionnaire's was rated as "unknown."

Patients' relatives were considered to have endometriosis when the disease had been diagnosed by laparoscopy or laparotomy. A genetic predisposition to endometriosis was assumed if, in addition to the endometriosis patient, one other female family member was found to suffer from endometriosis.

### Statistical analysis

Variables are described by frequencies and mean ± standard deviation (SD). Statistical analysis was performed in SPSS 15.0.1 for Windows (SPSS Inc, 1989-2006). Contingency tables and chi-square analysis were used in order to compare incidences between groups. The endometriosis incidences are presented as absolute risks (percentages); in order to compare the risks between groups, odds ratios were calculated. All values are given with a 95% confidence interval (95% CI).

Since exact information on the endometriosis-related medical history was not available for all relatives ("unknown" answers in the completed questionnaire), the familial incidence of endometriosis in first-degree relatives was calculated using two different approaches: (a) "real case analysis": all family members with insufficient information about presence of endometriosis were rated as non-endometriotic subjects; (b) "worst case analysis": all relatives of endometriosis patients with insufficient information were rated as affected by endometriosis. Since, the number of relatives with incomplete information about endometriosis-related health issues increased with the degree of relationship, a worst-case analysis was not performed for second- and higher-degree relatives; the incidence of relatives with endometriosis would have been inordinately high in the endometriosis group.

## Results

Patients in the endometriosis (n = 80) and the control group (n = 60) were 37.7 ± 6.2 and 45.9 ± 12.0 years of age at the time of the interview, respectively (p < 0.05). The total number of first-, second-, and third-degree relatives was 425 in the endometriosis group and 749 in the control group. The information about the presence of endometriosis was more readily available for relatives of those in the endometriosis group than for relatives of those the control group (325/749 [43.4%] vs. 239/425 [56.2%], p < 0.05).

In order to rule out a bias based on family size and the number of affected family members, the study collective was divided in two subgroups: families with four or less female family members who were affected; and families with more than four affected female family members. The incidence of endometriosis was 13.0% and 17.6%, respectively. This difference was statistically not significant (p < 0.05). Thus, it was assumed that family size had no major influence on the incidence of endometriosis.

General characteristics and information about the presence of symptoms associated with endometriosis are listed in Table 2.

**Table 2:** Characteristics of endometriosis patients (n = 80)

age at onset of endometriosis symptoms < 25 years	27 (33.8%)
severe endometriosis (rAFS^a ^IV)	21 (26.3%)
dysmenorrhea	66 (82.5%)
severe dysmenorrhea (V.A.S.** ≥ 7)	20 (25.0%)
chronic pelvic pain	16 (20.0%)
severe chronic pelvic pain (V.A.S.** ≥ 7)	14 (17.5%)
infertility	43 (53.8%)
pain on defecation	29 (36.3%)
severe pain on defecation (V.A.S.** ≥ 7)	21 (26.3%)
dyspareunia	13 (16.3%)
severe dyspareunia (V.A.S.** ≥ 7)	24 (30.0%)

^a ^rAFS = Revised American Fertility Society classification of endometriosis

** V.A.S. = Visual analog score

Table 3 gives an overview of the incidence of endometriosis in first-degree relatives of endometriosis patients and controls. In 5/136 (3.7%) and 8/134 (6.0%) first-degree relatives of endometriosis patients and the control group, respectively, information about the presence of endometriosis was not available (p = 0.554).

**Table 3:** Incidence of endometriosis-affected first-degree relatives of endometriosis patients and controls

	Real-case analysis			
**Relatives**	**Endometriosis group (n = 80)**	**Control group (n = 60)**	**Odds ratio** [95% CI]*	**P-value**

Mothers	5/80 (6.3%)	2/60 (3.3%)	1.93 [0.32; 14.98]	n.s.^+^

Daughters	0/9 (0%)	1/15 (6.2%)	-	n.s.^+^

Sisters	3/47 (6.4%)	1/59 (1.7%)	3.95 [0.35;100.59]	n.s.^+^

Total	8/136 (5.9%)	4/134 (3.0%)	2.03 [0.54; 8.25]	n.s.^+^

				

	**Worst-case analysis**			

**Relatives**	**Endometriosis group (n = 80)**	**Control group (n = 60)**	**Odds ratio** [95% CI]*	**P-value**

Mothers	9/80 (11.3%)	2/60 (3.3%)	3.67 [0.70; 25.68]	n.s.^+^

Daughters	0/9 (0%)	1/15 (6.2%)	-	n.s.^+^

Sisters	4/47 (8.5%)	1/59 (1.7%)	5.40 [0.55; 129.08]	n.s.^+^

Total	13/136 (9.6%)	4/134 (3.0%)	3.46 [1.01; 13.00]	0.042

* 95% CI = 95% confidence interval;^+ ^n.s. = not significant

By pooling all first-degree relatives, there was no significant difference between relatives of patients and controls: endometriosis was found in 8/136 (5.9%) first-degree relatives of patients and in 4/134 (3.0%) first-degree relatives of controls in the real-case analysis (p = 0.248). The worst-case analysis revealed a higher incidence of endometriosis in first-degree relatives of patients (9.6%; 13/136) than in first-degree relatives of controls (3.0%; 4/134) (p = 0.042).

Details of real-case analysis in second- and third-degree relatives are listed in Table 4. In the course of the interview, information about the presence of endometriosis was evaluated separately for maternal and paternal second- and third-degree relatives.

**Table 4:** Incidence of endometriosis-affected second- and third-degree relatives of endometriosis patients and controls

Relatives	Endometriosis group (n = 80)	Control group (n = 60)	Odds ratio [95% CI]*	P-value
Aunts	2/159 (1.3%)	1/127 (0.8%)	1.61 [0.11; 44.62]	n.s.^+^
Cousins	2/139 (1.4%)	1/134 (0.8%)	1.94 [0.14; 54.00]	n.s.^+^
Total	4/298 (1.3%)	2/261 (0.8%)	1.76 [0.28; 13.95]	n.s.^+^

* 95% CI = 95% confidence interval;^+ ^n.s. = not significant

Both cases of endometriosis in aunts and cousins of the control group were paternal relatives (2/2, 100%), whereas 3/4 affected second- and third-degree relatives (2 aunts and 1 cousin) of endometriosis patients were maternal relatives (3/4, 75.0%).

**Figure 1:**
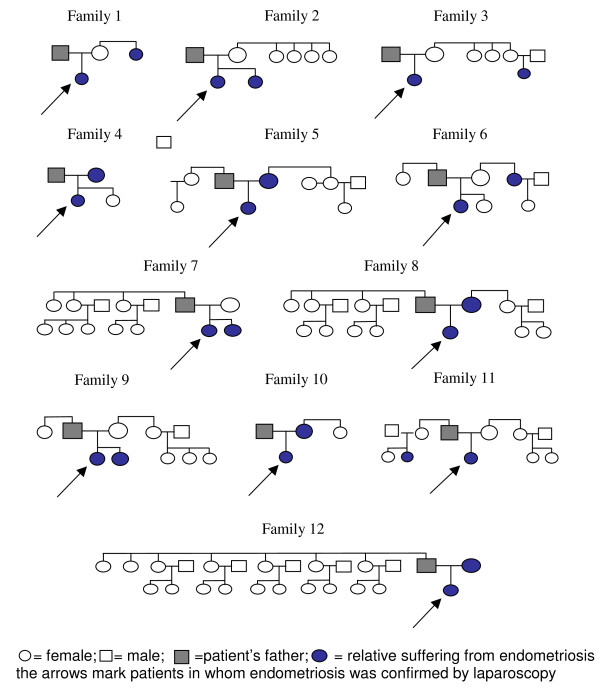
**Family tree of patients with a familial incidence of endometriosis**.

A total of 12/80 endometriosis patients (15.0%) with one relative suffering from endometriosis were found. The family trees of these cases with familial clustering are shown in Figure 1.

When comparing endometriosis characteristics between endometriosis patients with and without an incidence of familial endometriosis, no significant differences were found. Details are given in Table 5.

**Table 5:** Comparison of characteristics between endometriosis patients with and without familial clustering

	patients with a familial incidence (n = 12)	patients without a familia incidence (n = 68)	P-value
age at onset of endometriosis < 25 years	5/12 (41.7%)	22/68 (32.4%)	n.s.*
severe endometriosis (rAFS** IV)	5/12 (41.7%)	16/68 (23.5%)	n.s.*
dysmenorrhea	10/12 (83.3%)	56/68 (82.4%)	n.s.*
severe dysmenorrhea (V.A.S.*** ≥ 7)	4/12 (33.3%)	16/68 (23.5%)	n.s.*
chronic pelvic pain	3/12 (25.0%)	13/68 (19.1%)	n.s.*
severe chronic pelvic pain (V.A.S.*** ≥ 7)	4/12 (33.3%)	10/68 (14.7%)	n.s.*
infertility	4/12 (33.3%)	39/68 (57.4%)	n.s.*
pain on defecation	3/12 (25.0%)	26/68 (38.2%)	n.s.*
severe pain on defecation (V.A.S.*** ≥ 7)	8/12 (66.6%)	13/68 (19.1%)	n.s.*
dyspareunia	3/12 (25.0%)	10/68 (14.7%)	n.s.*
severe dyspareunia (V.A.S.*** ≥ 7)	4/12 (33.3%)	20/68 (29.4%)	n.s.*

* n.s. = not significant

** rAFS = Revised American Fertility Society classification of endometriosis

*** V.A.S. = Visual analog score

## Discussion

In our data, a tendency toward increased risk of endometriosis in first-degree relatives of endometriosis patients (5.9% vs. 3.0% in real-case analysis, odds ratio 2.03) was found. However, this tendency did not reach statistical significance (p < 0.05). This is in contrast to other studies that clearly demonstrate a stronger heritability [4,5,7-10]. Table 6 provides an overview of these research articles.

**Table 6:** Overview of research on the familial incidence of endometriosis in first-degree relatives

Study	Endometriosis group (absolute risk)	Control group (absolute risk)	Odds ratio [95% CI]*	P-value
Simpson et al. [4]	6.9%	0.9%	7.7 [1.7; 48.6]	< 0.005
Moen & Magnus [7]	3.9% - 4.8%	0.6% - 0.7%	7.2 [2.1; 24.3]	< 0.01
Coxhead & Thomas [9]	5.8%	0.8%	7.9 [1.5; 55.7]	< 0.01
Lamb et al. [5]	4.9%	~ 1.0%	~ 5.0	*unknown*
dos Reis et al. [8]	8.6%	0%	-	< 0.01
Nouri et al.	6.3%	3.3%	1.9 [0.3; 15.0]	n.s. *

* n.s. = not significant

The first publication about this topic reported a seven-fold increased risk for the development of endometriosis in first-degree relatives of endometriosis patients in contrast to a control group [4]. The absolute risk of endometriosis in patients' mothers and sisters was 8.1% and 5.8%, respectively. Similarly, a Norwegian study, conducted by Moen and Mangus [7], also showed a seven-fold greater risk for endometriosis in first-degree relatives of endometriosis patients. One study examining only relatives of patients with endometriosis reported even higher rates: The authors found that even 22% of patients' sisters of reproductive age and 16% of the mothers had a surgical diagnosis of endometriosis [11].

Considering the above-mentioned up to seven-fold increased risk for the development of endometriosis in first-degree relatives of endometriosis patients, as well as the life-time risk for endometriosis of about 10% in the general female population, one would expect a clearly increased rate of affected first-degree relatives of endometriosis patients. However, this was not the case in our study collective. Notably, patients of the control group were significantly older than the endometriosis patients (45.9 ± 12.0 vs. 37.7 ± 6. years) in our study collective. It could be that the age-factor influenced the results of our study and contributed to the only slightly increased risk for endometriosis in first-degree relatives of endometriosis patients. However, several probands of the non-endometriosis group belonged to an older generation. A detailed diagnostic work-up of endometriosis, which included diagnostic laparoscopy, was not routine then. Thus, several cases of endometriosis may have remained undiagnosed. On the other hand, the greater age of the controls increased the possibility of having first-degree relatives, especially daughters, thus potentially increasing the number of first-degree relatives with endometriosis. However, as mentioned in the Results section, we ruled out the influence of family size on the incidence of endometriosis. The difference in patients' and probands' age was less than a whole generation. Thus, we consider it unlikely that age had any influence on the incidence of endometriosis in relatives.

Another factor that may have contributed to the moderate difference in endometriosis incidence between relatives of the patient and the control groups might be the patient selection method in our study. For the patient group, we chose endometriosis patients who presented at our department with infertility rather than chronic pelvic pain. The control group, however, consisted of patients who had undergone laparoscopy for reasons other than infertility. Nonetheless, we compared endometriosis patients to patients without endometriosis and our study collective might represent the general population more accurately than a study collective including patients with chronic pelvic pain.

In the worst-case analysis, we rated all first-degree family members of endometriosis patients for whom the medical history was unknown as "affected by endometriosis." Thus, we found a significantly higher endometriosis incidence in patients' relatives. However, the calculated odds ratio of 3.5 was still lower than that reported in the literature.

Taking all these considerations together, we consider our results sound. Thus, the familial incidence of endometriosis demonstrated in our study supports the theory that a genetic predisposition is only a contributing factor for the development of endometriosis. As known from twin-studies, endometriosis seems to have a genetic basis: increased concordance rates for endometriosis of 75% and 88% in monozygous twins have been demonstrated [12]. However, since we found a moderately increased risk for first-degree relatives, with an odds ratio of about 2, genetics may play a less important role than previously thought. Thus, the cause of the development of endometriosis is likely more multifactorial. Life-style, the use of hormonal contraceptives, nutrition, age at first and/or last pregnancy, and other factors, may contribute to the disease. Furthermore, it might be argued that in families with affected members there is a greater awareness about endometriosis, which might lead to a more invasive diagnostic work-up, including diagnostic laparoscopy, which has been suggested [13]. This might also contribute to the higher rates of affected relatives of endometriosis patients in other studies.

Most of the reported studies have not evaluated the incidence of endometriosis in second- and third-degree relatives, since the patients' information on the medical history of these relatives was considered unreliable. Notably, Lamb et al. reported an endometriosis incidence (absolute risk) in second-degree relatives of endometriosis patients, specifically, the patients' aunts and grandmothers, of 1.9% [5]. Despite the fact that we did not evaluate the rate of endometriosis in patients' grandmothers in our study collective, we found a similar endometriosis incidence in second-degree relatives of 1.3%. In 1999, a Brazilian study evaluated the risk for endometriosis in third-degree relatives, for the first time, and reported an incidence of 2.4% [8]. With regard to second- and third-degree relatives, there were no differences in the endometriosis incidences between the patient and the control groups. Interestingly, 3/4 cases of endometriosis in second- and third-degree relatives of the endometriosis group were maternal relatives in contrast to 0/2 in the control group. This finding is clearly consistent with a genetic basis for the incidence of familial endometriosis.

Several studies have shown higher severity scores of endometriosis-associated symptoms in patients with a family history of endometriosis [14]. In our study, endometriosis patients with a greater familial incidence of endometriosis than other endometriosis patients suffered from severe endometriosis (41.7% vs. 23.5%), severe chronic pelvic pain (33.3% vs 14.7%), and severe pain on defecation (66.6% vs. 19.1%). However, this may be due to the low sample size, and thus, these differences were not statistically significant. In addition, more patients with a greater familial incidence of endometriosis showed an early age at onset of < 25 years (41.7% vs. 32.4%). These findings all support the theory of a polygenous hereditary system.

The fact that all patients from the control group were proven laparoscopically not to be affected by endometriosis is a major strength of our study. This is in contrast to other studies that did not include patients for whom endometriosis had been ruled out by laparoscopy as control groups [4,8,9,15]. The sub-analysis of patients with a greater incidence of endometriosis versus patients without a familial incidence of endometriosis seems under-powered. A surgical diagnosis of the disease was not performed in the relatives and, then, several cases of endometriosis may have remained undiagnosed. We consider this a limitation of the study.

Several genetic studies aiming to discover the genes involved the susceptibility to endometriosis are ongoing. Future genomic studies may lead to new non-invasive diagnostic strategies as well as possible new therapies. These data will hopefully improve our understanding of the etiology of endometriosis [16].

## Conclusion

In summary, our case-control study demonstrates a trend toward greater familial incidences of endometriosis. In contrast to the literature, we found a less dramatic increase in familial risk for the development of endometriosis.

## Competing interests

The authors declare that they have no competing interests.

## Authors' contributions

KNO performed analysis and reporting of the study. BKZ performed acquisition of the data presented in Tables 1, 2, 3, 4, 5 and Figure 1. JOH performed acquisition of the data presented in Tables 6 and analysis. JCH organized the program. RWL performed revision of the manuscript. All authors read and approved the final manuscript.
